# Incidental Intraoperatively Detected Choledocholithiasis: A General Surgeon’s Approach to Management

**DOI:** 10.7759/cureus.47634

**Published:** 2023-10-25

**Authors:** Jun Guang Kendric Tan, Jessica O'Sullivan, Ruwan Wijesuriya

**Affiliations:** 1 General Surgery, St John of God Midland Public and Private Hospitals, Perth, AUS

**Keywords:** cholelithiasis, intra-operative cholangiogram, hepatobiliary, choledocholithiasis, cholecystectomy

## Abstract

Background

Up to 15% of patients with cholelithiasis have choledocholithiasis, with almost 10% not detected pre-operatively. Our study aims to quantify the prevalence of incidental choledocholithiasis during routine intra-operative cholangiogram (IOC), identify the best management pathway, and identify reliable pre-operative factors to predict choledocholithiasis.

Methods

We conducted a single-centre, retrospective cohort study at St John of God Midland Hospital in Western Australia, Perth, on 880 consecutive patients who underwent cholecystectomies performed by 15 surgeons between January 2, 2020, and December 30, 2021.

Results

The overall choledocholithiasis rates were 10.6% (93), with 4.0% (35) diagnosed pre-operatively and 6.6% (58) diagnosed during IOC. In all, 50% of incidental choledocholithiasis during IOC were managed with hyoscine butylbromide, with a 55.2% success rate; 22.4% of patients received octreotide, with a 61.5% success rate; and 8.6% of patients underwent trans-cystic bile duct exploration (TCBE) and 8.6% underwent postoperative endoscopic retrograde cholangiopancreatography (ERCP), both with 100% success rates. Choledocholithiasis most commonly presents with gallstone pancreatitis, with a median aspartate aminotransferase (AST) level 7.2 times and alanine transaminase (ALT) level 7.8 times higher than those of patients without choledocholithiasis. Magnetic resonance cholangiopancreatography (MRCP) was the most sensitive in identifying choledocholithiasis with a 66.7% pickup rate. The median common bile duct (CBD) diameter on ultrasound was 8 mm, computerised tomography scans were 11 mm, and MRCP was 9 mm.

Conclusion

One in 10 cholecystectomies will be complicated with choledocholithiasis, and over half will be incidentally diagnosed during routine IOC. We propose IOC in all cases and hyoscine butylbromide, octreotide, and saline flushes as first-line treatment; if unsuccessful, TCBE is performed. Gallstone pancreatitis, markedly elevated AST/ALT, and imaging showing CBD ≥8 mm may serve as early predictors of choledocholithiasis.

## Introduction

Gallbladder pathology is increasingly common in Australia, with over 1 million hospital admissions over a 15-year period (2004-2019), and 73% of these cases required cholecystectomy, equating to 197 cholecystectomies per 100,000 population in Australia [1]. Cholelithiasis is the most common gallbladder-associated condition in Australia, accounting for 86.7% of all admissions [1]. While most patients with cholelithiasis are asymptomatic, 10-20% will develop symptoms within 20 years of diagnosis [2]. These symptoms can range in severity from uncomplicated biliary colic to ascending cholangitis. It is estimated that choledocholithiasis is present in up to 15% of patients with cholelithiasis [3]. Choledocholithiasis can manifest as a range of clinical presentations, including stone-related colicky pain, gallstone pancreatitis, or cholangitis. Various management pathways have been suggested by different committees, with key intervention periods being pre-operative, intra-operative, and post-operative. Cholangitis, elevated bilirubin levels, and a dilated common bile duct (CBD) on imaging have been proposed as predictive factors for choledocholithiasis and should prompt further workup [4,5]. A common clinical challenge is presented by patients with biochemically obstructed liver function tests but no imaging findings of choledocholithiasis. This often leads to a cholecystectomy and intra-operative cholangiogram (IOC) to confirm the presence of intra-ductal gallstones. If an incidental stone is confirmed on IOC, various methods of retrieval are available, each with varying success rates. Pharmacological agents can be administered intra-operatively, in conjunction with biliary tree flushing, to flush stones out of the CBD. If anatomical factors allow for it, trans-cystic bile duct exploration (TCBE) can be performed. If all intra-operative measures fail, post-operative endoscopic retrograde cholangiopancreatography (ERCP) often has high success rates [6].

The primary aim of this study is to evaluate the management pathways for incidental choledocholithiasis discovered during IOC, including intra-operative strategies with pharmacological agents and flushing, TCBE, or ERCP.

Secondary aims include identifying reliable pre-operative factors to predict choledocholithiasis based on clinical, radiological, and biochemical factors. Given the varying adoption of the practice of routine IOC, with rates ranging from 20% to 82% [7], we aim to define the frequency of IOC showing stones when there is no pre-operative suggestion on radiological grounds, exploring the benefits of routine IOC. Additionally, we will analyse the indications and histopathology of all cholecystectomies.

## Materials and methods

Study design

This retrospective cohort study was conducted at St John of God (SJOG) Midland Public and Private Hospital, a 307-bed public hospital in Western Australia, Perth. We identified 880 consecutive patients who underwent cholecystectomies performed by 15 surgeons between January 2, 2020, and December 30, 2021, based on medical records. ERCP procedures were conducted either at St John of God Midland Private Hospital or Royal Perth Hospital (RPH), a 450-bed teaching tertiary hospital. Exclusion criteria included incomplete entries and individuals under 18 years old. All surgeons involved are either certified by the Royal Australasian College of Surgeons (RACS) or supervised by a surgeon recognised by RACS. No patients were specifically recruited for our study; instead, our analysis was solely based on auditing clinical notes, with a focus on biochemical and radiological findings, management methods, clinical outcomes, and predictive factors. We employed convenience sampling from January 1, 2018, to December 31, 2022.

Data collection

Clinical notes from SJOG Midland Hospital and RPH were reviewed by two investigators. The collected information encompassed patient demographics, details of clinical presentation, operative notes, imaging reports, histopathological results, and ERCP reports. Indications for cholecystectomy were extracted from both operation notes and patient admission records. Operation reports were carefully examined for the results of intra-operative cholangiograms. Anaesthetic records were reviewed to determine the pharmaceutical agents administered. Imaging reports from the local radiological provider were methodically analysed for anatomical details concerning the gallbladder and CBD. Pathology reports were reviewed for inflammatory markers and liver function tests, with the highest pre-operative value during the current admission being recorded.

Statistical analysis

Comparative statistics were performed with two-tailed Fisher’s exact test and logistic regression using GNU PSPP version 1.6.2. A p-value of <0.05 was considered statistically significant.

Ethics

Local ethics approval was obtained through the St John of God Health Care Human Research Ethics Committee and the Royal Perth Hospital Human Research Ethics Committee. All data were de-identified, password protected, and kept on secure servers.

## Results

A total of 880 patients had cholecystectomies. The median age of all patients was 50 (18-92) years, with a female-to-male ratio of 2.6:1. Eighty-seven percent of patients had routine IOC performed. Of the 114 (13%) patients who did not have IOC performed, none were complicated with choledocholithiasis.

**Figure 1:**
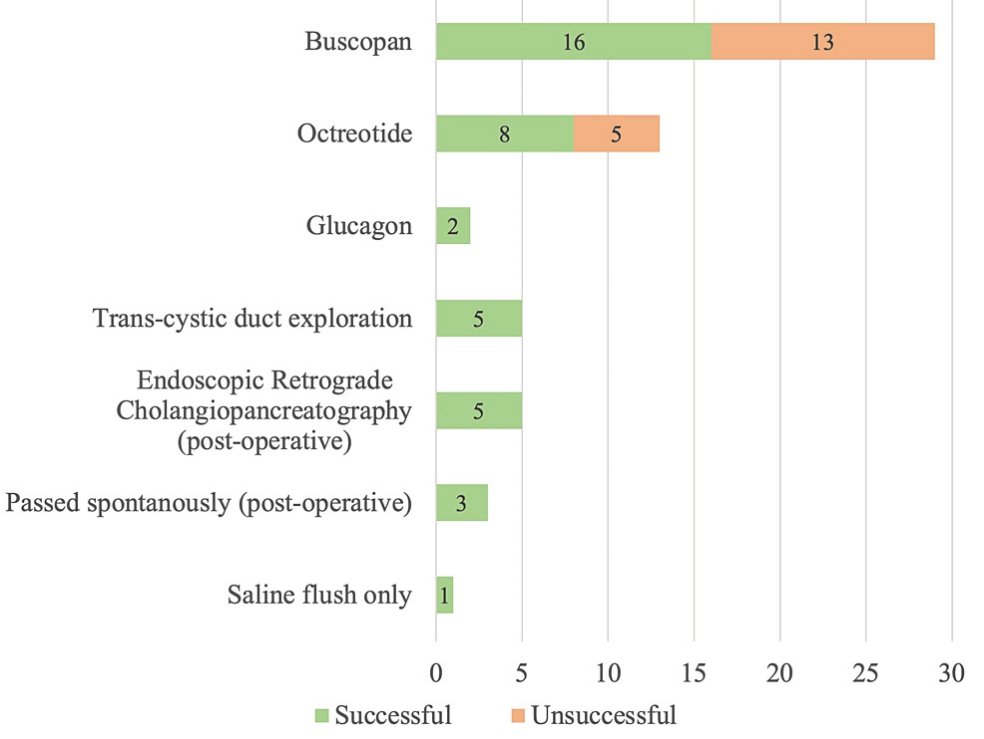
Management of choledocholithiasis diagnosed during intra-operative cholangiogram (n = 58)

Ninety-three of 880 (10.6%) patients who underwent cholecystectomy were diagnosed with choledocholithiasis, with 58 (6.6%) diagnosed incidentally during IOC. Of these 58 patients, 29 (50.0%) were managed with an average of 22 ml of Buscopan (5-30 ml), with a success rate of 55.2%. Thirteen (20.6%) patients were managed with 100 µm of Octreotide, with a success rate of 61.5%. Two (3.2%) patients were managed with 1 mg of Glucagon, with a 100% success rate. TCBE was attempted in 5 (8.6%) patients, with a 100% success rate. Five (8.6%) patients had post-operative ERCP with a 100% success rate. Success was confirmed with intra-operative cholangiogram showing no filling defects and resolution of liver function tests post-operatively. There were no recorded side effects from the administration of these pharmacological agents. Three (4.8%) patients had stones pass spontaneously post-operatively with no intervention and 1 (1.6%) patient was successfully managed with saline flushes alone. This is presented in Figure 1.

**Figure 2:**
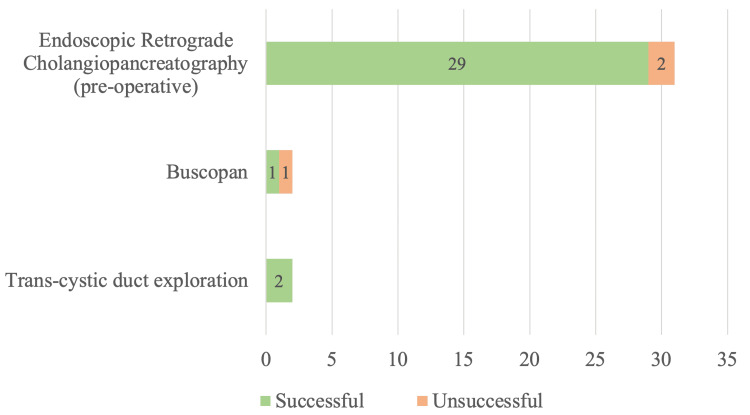
Management of choledocholithiasis diagnosed before cholecystectomy (n = 35)

Thirty-five (4.0%) patients were diagnosed with choledocholithiasis before cholecystectomy via clinical and imaging means. Of these, 31 (88.6%) patients were managed with pre-operative ERCP, with a success rate of 93.5%. Four (11.4%) patients had intra-operative interventions, with 2 (5.7%) having intravenous Buscopan and saline flushes via CBD with a 50% success rate, and 2 (5.7%) having planned TCBE with a 100% success rate. This is presented in Figure 2.

**Figure 3:**
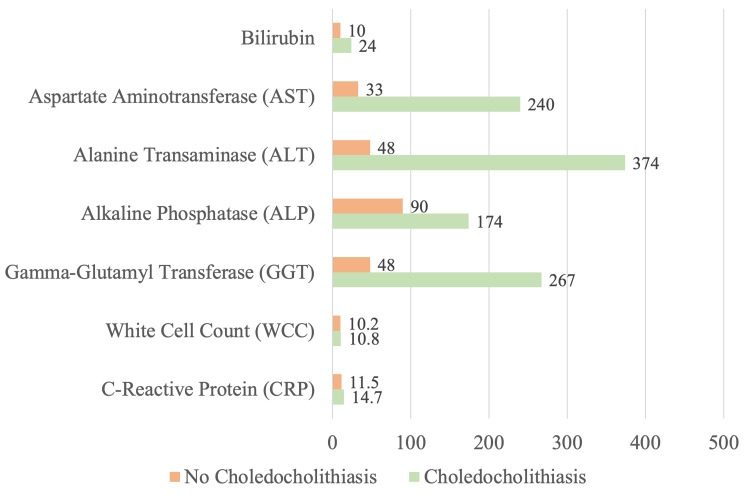
Serum biochemistry markers (median) of patients with choledocholithiasis versus without choledocholithiasis

Of the 93 (10.6%) patients who underwent cholecystectomy with pre-operative imaging or IOC-proven choledocholithiasis, 26 (28.0%) presented with gallstone pancreatitis, and 25 (26.9%) presented with choledocholithiasis as the primary diagnosis. Twenty-three (24.7%) presented with biliary colic and 19 (20.4%) with cholecystitis, where choledocholithiasis was an incidental finding. Five (5.4%) patients presented with clinical jaundice. The median bilirubin level was 24 umol/L (2-165), aspartate aminotransferase (AST) was 240 U/L (13-1610), alanine transaminase (ALT) was 374 U/L (18-1918), alkaline phosphatase (ALP) was 174 U/L (48-1120), gamma-glutamyl transferase (GGT) was 267 U/L (3-1239), white cell count (WCC) was 10.8 x 10^9^/L (5.6-21) and C-reactive protein (CRP) was 14.7 mg/L (0.9-443.8). Figure 3 illustrates the comparative median biochemical values of patients with choledocholithiasis versus those without choledocholithiasis.

Analysing pre-operative imaging modalities, 76 (81.7%) patients had an abdominal ultrasound (USS) performed, with a median CBD diameter of 8 mm (3-14 mm) and 11 (14.5%) showing choledocholithiasis. Twenty-three (24.7%) patients had computerised tomography scans, with a median CBD diameter of 11 mm (8-13 mm) and 2 (8.7%) showing choledocholithiasis. Twenty-one (22.6%) patients had magnetic resonance cholangiopancreatography (MRCP) scans, with a median CBD diameter of 9 mm (7-14 mm) and 14 (66.7%) showing choledocholithiasis.

Of the remaining 787 (89.4%) patients who underwent cholecystectomy without choledocholithiasis, 394 (50.1%) presented with biliary colic, 305 (38.8%) with cholecystitis, 59 (7.5%) with gallstone pancreatitis, 6 (0.8%) with clinical choledocholithiasis, 10 (1.3%) with gallbladder dysfunction, 9 (1.1%) with gallbladder lesion(s) and 4 (0.5%) were performed during bariatric surgery.

**Figure 4:**
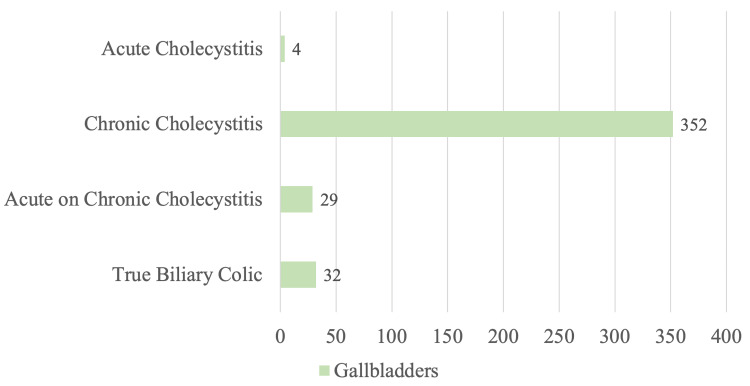
Histological findings of cholecystectomies performed for clinical biliary colic

Out of the 417 patients who presented with clinical biliary colic, 385 (92.3%) had histological evidence of cholecystitis. Four (1.0%) patients had acute cholecystitis, 352 (84.4%) patients had chronic cholecystitis, 29 (7.0%) patients had acute or chronic cholecystitis and 32 (7.7%) had true biliary colic. This is presented in Figure 4.

## Discussion

Optimal method of managing choledocholithiasis

In our centre, management of choledocholithiasis is heavily dependent on the timing of the diagnosis, in particular pre-operatively or post-operatively. We believe it is beneficial to diagnose choledocholithiasis prior to performing cholecystectomy as this gives us the option to plan for a pre-operative ERCP if indicated. This relieves intra-biliary pressure, lowering the risk of cystic stump leak and reducing the time pressure for an extended procedure on a busy emergency theatre list [8].

The choice of pre-operative ERCP or intra-operative techniques is also dependent on the patient’s age. Endoscopic sphincterotomy impairs the function of the biliary sphincter, allowing for chronic reflux of digestive enzymes and bacteria into the biliary tree. Chronic inflammation has been associated with epithelial hyperplasia, atypism, and subsequent malignancy. Hence, we favour intra-operative techniques to treat choledocholithiasis in the younger population rather than ERCP [9,10].

In our study population, which reflects local practice, 31 of 35 (88.6%) pre-operatively diagnosed choledocholithiasis cases were managed with pre-operative ERCP, with a high success rate of 93.5%. The remaining 4 of 35 (11.4%) cases underwent cholecystectomy with planned TCBE, of which 50% had CBD cleared with Buscopan and saline flushes alone and 50% required progression to TCBE. Clinical reasons to choose intra-operative management of choledocholithiasis rather than pre-operative ERCP included the presence of multiple CBD stones and a widely dilated CBD, in which there is a high chance of further stones migrating into the CBD in the time between ERCP clearance and cholecystectomy. Other reasons included small distal stones that will likely respond to intra-operative CBD flushing or a young patient age.

On the other hand, incidental choledocholithiasis diagnosed during routine IOC during cholecystectomy was usually managed with pharmacological agents (Buscopan/Octreotide/Glucagon) to decrease sphincter of Oddi’s pressure and subsequent saline flushes into the CBD via cholangiogram catheter. Although the performance of Buscopan versus Octreotide was not statistically significant at p=0.75, Octreotide appears to have higher success rates than Buscopan in clearing CBD stones.

TCBE is highly dependent on surgeon expertise and equipment availability [11]. Only 5 of 58 (8.6%) cases underwent TCBE but with a 100% success rate. We noted a case where pre-operative ERCP failed due to difficult biliary cannulation, with subsequent success with TCBE. Patient selection such as the number of stones, size of stones, and routine IOC prior to identifying cystic duct anatomical variations contributes to a successful TCBE [12].

Pre-operative factors to predict choledocholithiasis

Gallstone pancreatitis (28.0%) and imaging-proven choledocholithiasis (26.9%) were the most common presentations for choledocholithiasis. Pancreatitis was defined as per the 2012 Revised Atlanta Classification of Acute Pancreatitis [13]. This is no surprise as the etiology of gallstone pancreatitis stems from distal biliary obstruction by gallstones. Jaundice was noted in the minority (5.4%).

We note liver function tests to be markedly deranged for patients with choledocholithiasis, with the median values of bilirubin 2.4 times higher, AST 7.2 times higher, ALT 7.8 times higher, ALP 1.2 times higher, and GGT 5.6 times higher than in patients without choledocholithiasis. Is it interesting to note that comparing patients with choledocholithiasis and without choledocholithiasis, the median AST and ALT values which are traditionally markers for hepatocellular injury were deranged to a much larger extent (7.2x-7.8x) than ALP and GGT (1.2x-5.6x), which usually demonstrates biliary epithelial damage. This pattern has been seen in other studies [14,15] with a suggestion that acute biliary obstruction causing hepatocyte necrosis is the underlying pathophysiological mechanism [16]. Logistic regression was used to calculate predictive probability for each value, which showed that raised bilirubin was the only strong predictor for choledocholithiasis (p = 0.011). In any case, clinicians must recognise the validity of this discrepancy, considering the clinical picture before interpreting liver function tests, thus preventing unnecessary investigations of other primary liver diseases.

MRCP was the most sensitive in identifying choledocholithiasis, picking up 14 of 21 cases (66.7%). However, it is resource-intensive and may not be readily available, with only 21 of 93 patients undergoing it (22.6%). Targeted ultrasonography is easily available, non-invasive, and quick to perform. Seventy-six of 93 patients were investigated with abdominal ultrasound (81.7%), making it the most common imaging modality. It is also the second most sensitive for choledocholithiasis, identifying 11 of 76 (14.5%) cases. Twenty-three of 93 (24.7%) patients with choledocholithiasis had pre-operative CT scans as part of the initial workup for undifferentiated abdominal pain. As most gallstones are radiolucent, it is not surprising that CT scans were not sensitive in diagnosing choledocholithiasis, picking up only 2 of 23 (8.7%) cases. What we can rely on are secondary signs of CBD dilatation. For patients with choledocholithiasis, the median CBD diameter on USS was 8 mm, that on CT scans was 11 mm, and that on MRCP was 9 mm, making a CBD diameter greater than 8 mm a relatively reliable indicator of choledocholithiasis.

Frequency of incidental choledocholithiasis on IOC

The 2010 Society of American Gastrointestinal and Endoscopic Surgeons (SAGES) guidelines for laparoscopic biliary tract surgery give a level 2 recommendation for the use of routine IOC during laparoscopic cholecystectomy, citing a lack of reliable algorithms to guide a selective policy [17]. The average choledocholithiasis on routine intraoperative cholangiogram is 2-12%, with as many as 10% not detected pre-operatively [18-20]. These are consistent with our results, with 6.6% of all patients undergoing cholecystectomies having incidental choledocholithiasis during routine IOC.

Even with predictive markers and advanced pre-operative imaging, more than 60% of choledocholithiasis diagnosed were incidental during routine IOC. It is our centre’s practice to perform routine IOC during laparoscopic cholecystectomies. The benefits of this include not only identifying the patients who may develop complications of post-operative choledocholithiasis (e.g., cystic stump blowout, biliary peritonitis, cholangitis), clarifying biliary anatomy and preventing CBD injuries but also developing the skills necessary for progression to TCBE and more specialised interventions. Although routine IOC has been criticised for its false positive rates resulting in unnecessary procedures and their associated morbidity, increased cost, and operative duration, we believe the benefits outweigh the potential risks [21].

Indication for cholecystectomies/open conversion rates

Biliary colic is the top indication for a cholecystectomy (47.4%). This is followed by cholecystitis (36.8%), gallstone pancreatitis (9.7%), choledocholithiasis (3.5%), gallbladder dysfunction (1.1%), gallbladder polyp/lesion (1%), and bariatric surgery (0.5%).

It was interesting to note that 92.3% of patients diagnosed clinically with biliary colic as the indication for cholecystectomy had some form of inflammation on histology. The most common was chronic cholecystitis (84.4%), followed by acute or chronic cholecystitis (7.0%). Only 7.7% had true biliary colic with no histological inflammation. This suggests that even a transient episode of biliary colic can cause histological inflammation not appreciable on diagnostic imaging.

Our series recorded an open conversion rate of 1.7%, well below the rates of 2-15% quoted in the literature [17,22]. The majority of open conversions were for gallbladder empyema or extensive adhesions for previous open surgeries, with only one case for bleeding control.

## Conclusions

Almost 1 in 10 cholecystectomies will be complicated with choledocholithiasis. Our centre prefers to decompress the biliary tree with pre-operative ERCP prior to a cholecystectomy but not all choledocholithiasis are picked up pre-operatively. Over half of all choledocholithiasis will be incidentally diagnosed during routine IOC, Hence, we propose intra-operative cholangiogram in all cases, a combination of intra-operative hyoscine butylbromide, octreotide, and saline flushes as first-line treatment for choledocholithiasis due to its high success rate and lower complication profile. If this fails, TCBE is highly effective, and if unsuccessful, the next step would be post-operative ERCP.

To facilitate early diagnosis of choledocholithiasis, we identified certain predictive factors, including evidence of gallstone pancreatitis, markedly elevated LFT and a combination of routine USS and selective MRCP showing CBD dilatation of more than 8 mm. Once diagnosed, pre-operative ERCP and planned TCBE have high success rates.
